# A giant umbilical cord: Benign finding or surgical emergency?

**DOI:** 10.1002/ccr3.2663

**Published:** 2020-01-20

**Authors:** Veronica Mugarab Samedi, Grant Miller, Edouard Saade, Kaarthigeyan Kalaniti

**Affiliations:** ^1^ University of Saskatchewan Saskatoon SK Canada; ^2^ Department of Pediatrics Royal University Hospital Saskatoon SK Canada

**Keywords:** congenital anomaly, umbilical‐urachal cyst, Urachus

## Abstract

Congenital urachal anomalies are usually asymptomatic. However, infection or obstruction of urachal remnant may result in serious complications. The giant umbilical cords with suspected internal communications could contain remnants and require surgical exploration. With timely recognition and surgical intervention, the outcome is generally good.

## Graphical abstract

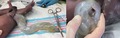

A boy (twin‐A) was born at 36 weeks to a primigravida mother after an uneventful pregnancy. After delivery, the baby's umbilical cord was noted to be bulky and gelatinous (Figure 1A) with a firmreddish sinus at the base (Figure 1B). There were no other dysmorphic features and no discharge from the umbilicus. His twin sister's examination was unremarkable.

**Figure 1:**
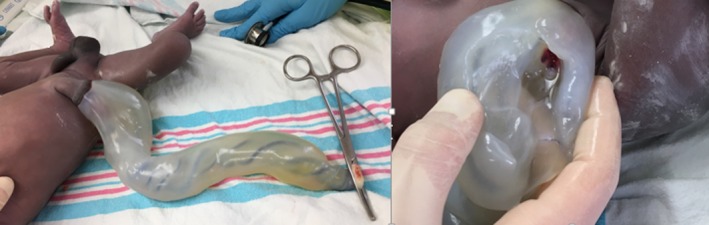
A, Shows the bulky and gelatinous umbilical cord and B, Shows a reddish sinus at the base of umbilical cord

As the sinus had outside communication, surgical exploration was done, which showed an abnormal tissue coming through the umbilicus cord extending to the bladder. Histopathology revealed urothelial‐lined tube surrounded by bundles of smooth muscle and umbilical cord that was consistent with umbilical‐urachal cyst remnant. Umbilical approach to sinus was used for resection of remnant, and postoperative course was uneventful. Now, the patient is 9 months old and doing very well.

A giant umbilical cord is an unusual finding in a newborn and usually associated with vascular or uracil anomalies. Congenital urachal anomalies are twice as common in male population, and majority of these patients are asymptomatic.^1^, ^2^ However, in neonates, infection or associated obstruction of the lower urinary tract may result in umbilical‐urinary fistulas.^2^ With timely surgical intervention, the outcome is generally good.^2^

## CONFLICT OF INTEREST

None declared.

## AUTHOR CONTRIBUTIONS

Dr V. Mugarab‐Samedi: conducted the literature review and drafted the manuscript. Dr G. Miller and Dr K.Kalaniti: assisted with the editing of manuscript. E. Saade: collected data, obtained consent, and contributed to the writing with commentary. All authors: revised the manuscript critically and gave approval for the final version to be published.
